# Recurrence of glioblastoma after radio-chemotherapy is associated with an angiogenic switch to the CXCL12-CXCR4 pathway

**DOI:** 10.18632/oncotarget.3256

**Published:** 2015-03-25

**Authors:** Emeline Tabouret, Aurelie Tchoghandjian, Emilie Denicolai, Christine Delfino, Philippe Metellus, Thomas Graillon, Celine Boucard, Isabelle Nanni, Laetitia Padovani, L'Houcine Ouafik, Dominique Figarella-Branger, Olivier Chinot

**Affiliations:** ^1^ Aix-Marseille Univ, CRO2, UMR 911, Marseille 13284, France; ^2^ APHM, Timone Hospital, Department of Neuro-Oncology, Marseille 13005, France; ^3^ APHM, Timone Hospital, Department of Neuro-Surgery, Marseille 13005, France; ^4^ APHM, North Hospital, Transfer Laboratory, Marseille 13015, France; ^5^ APHM, Timone Hospital, Department of Radiotherapy, Marseille 13005, France; ^6^ APHM, Timone Hospital, Department of Anatomopathology, Marseille 13005, France

**Keywords:** angiogenesis, glioblastoma, paired, switch

## Abstract

Angiogenesis is one of the key features of glioblastoma (GBM). Our objective was to explore the potential changes of angiogenic factors in GBM between initial diagnosis and recurrence after radiotherapy-temozolomide (RT/TMZ). Paired frozen tumors from both initial and recurrent surgery were available for 29 patients. Screening of genes expressions related to angiogenesis was performed using RT- PCR arrays on 10 first patients. Next, RNA expressions of the selected genes were analyzed on all samples. Protein expression was examined by immunohistochemistry. The anti-tumor effect of AMD3100 (anti-CXCR4) was tested in GBM explants. In the screening step, the initial-recurrence expression changes contributed to a selection of seven genes (*VEGFA, VEGFR2, VEGFR1, CXCL12, CXCR4, uPA HIF1α).* By quantitative RT-PCR, RNA expressions of *CXCR4* (*p* = 0.029) and *CXCL12* (*p* = 0.107) were increased while expressions of *HIF1α* (*p* = 0.009) and *VEGFR2* (*p* = 0.081) were decreased at recurrence. Similarly, CXCL12 protein expression tended to increase (*p* = 0.096) while VEGFR2 staining was decreased (*p* = 0.004) at recurrence. An increase of anti-tumoral effect was observed with the combination of AMD3100 and RT/TMZ versus RT/TMZ alone in GB explants. Recurrence of GB after chemo-radiation could be associated with a switch of angiogenic pattern from *VEGFR2-HIF1α* to *CXCL12-CXCR4* pathway, leading to new perspectives in angiogenic treatment

## INTRODUCTION

Glioblastoma (GBM) is the most frequently occurring primary brain tumor among adults and is one of the most lethal tumors. To date, recurrence is inevitable. Standard of care (SOC) in the first-line setting is based on the association of radiotherapy and concomitant and adjuvant temozolomide [1]. Biologically, GBMs are characterized by extensive angiogenesis, and vascular endothelial growth factor (VEGF) expression has been reported to be one of the highest among cancers [2]. Blocking this factor with a specific monoclonal antibody inhibited GBM growth *in vivo* [3], leading to interest in the evaluation of anti-angiogenic therapy in GBM patients. Recently, bevacizumab has been found to exhibit remarkable activity for patients with recurrent GBM, with response rates ranging from 30% to 50% [4, 5]. These results compare favorably with chemotherapy alone with regard to recurrence [6]. Bevacizumab was investigated in the first-line setting in two large randomized phase III trials (AVAglio [7] and RTOG 0825 [8]). In these trials, progression-free survival (PFS) was 3–4 months longer for patients receiving bevacizumab in addition to SOC compared with placebo in addition to SOC, while no difference in overall survival (OS) was observed. Of note, a cross-over effect may have partially contributed to this finding because 30%–50% of patients in the control arm received bevacizumab at recurrence. These results highlight the difficulty of determining the optimal timing of bevacizumab treatment.

To date, no convincing data have identified a robust predictive biomarker of response or survival for bevacizumab in various cancers treated with this agent. In a phase II uncontrolled trial that evaluated bevacizumab in patients with recurrent high-grade astrocytoma, an exploratory analysis suggest that high VEGF expression, as assessed in samples of the initial tumor, was associated with an increased likelihood of radiographic response, but not with survival at the time of recurrence [9]. However, the pattern of VEGF expression over the course of the disease is unknown. Determination of the expression profile of angiogenic factors at recurrence compared with their expression at initial diagnosis may identify a specific progression pathway, enabling the identification of new therapeutic targets. Previous studies have compared specific biological profiles between initial and recurrent GBM samples, including O(6)-methylguanine-DNA methyltransferase (*MGMT*) promoter methylation status, expression of proliferative markers or DNA copy number [10–13]; however, the aim of our study was to focus on putative changes in angiogenic pathways, excluding other potential molecular changes. The objective of our study was to compare the expression profile of angiogenic factors at the time of initial diagnosis to that observed at the time of recurrence in paired samples of GBM patients receiving radio-chemotherapy with no bevacizumab as first-line treatment.

## RESULTS

### Patient characteristics (Table 1)

Twenty-nine patients with a median age of 57.1 years (range 37.2–74.1 years) were enrolled in the present study between January 2003 and November 2009. The majority of the patients presented with a good performance status. All patients were treated with radio-chemotherapy as first-line treatment. At recurrence, all patients received Gliadel^®^ implants as part of the surgical treatment, while 14 patients (48%) also received bevacizumab as salvage treatment for subsequent recurrence after reoperation during the course of their disease. Median delay between the initial and the second surgery at recurrence was 10.9 months (range, 6.6–38.1), avoiding potential pseudo-progression for these patients. All patients were *IDH1/2* negative. The *MGMT* promoter was methylated in 6/27 (22%) patients.

**Table 1:** Patient characteristics

Characteristics	*N* = 29	%
**Initial diagnosis**		
**Age**	57, 1 (37, 2–74, 1)	
**Gender (M / W)**	18M / 11W	62% / 38%
**KPS (median)**	80	
60	1	*4%*
70	11	*38%*
80	14	*48%*
90–100	3	*10%*
**MMS**		
Normal	22	*85%*
Abnormal	4	*15%*
**Steroids**	20	*71%*
**RPA classification**		
III	4	*15%*
IV	19	*73%*
V–VI	3	*12%*
**Type of surgery**		
Gross total resection	28	*96%*
Other	1	*4%*
**MGMT status**		
Methylated / Unmethylated	6 / 21	*22% / 78%*
**IDH 1/2 mutation**	0	*0%*
**First line treatment**		
Radiotherapy and temozolomide	27	*93%*
Radiotherapy and BCNU	2	*7%*
**Recurrence**		
**Age**	58 (38, 2–75, 7)	
**KPS (median)**	70	
60	6	*21%*
70	15	*52%*
80	7	*24%*
90	1	*3%*
**Steroids**	24	*86%*
**Treatment at progression**		
Gliadel	29	*100%*
Bevacizumab	14	*48%*
**Total number of lines**		
2	6	*21%*
3	14	*48%*
4 or 5	9	*31%*

KPS: Karnofsky Performans Satus; MMS: MiniMental Status; RPA: Recursive Partitioning Analysis

### RT^2^ polymerase chain reaction (PCR) arrays (Figure 1A, 1B, 1C and 1D)

**Figure 1:**
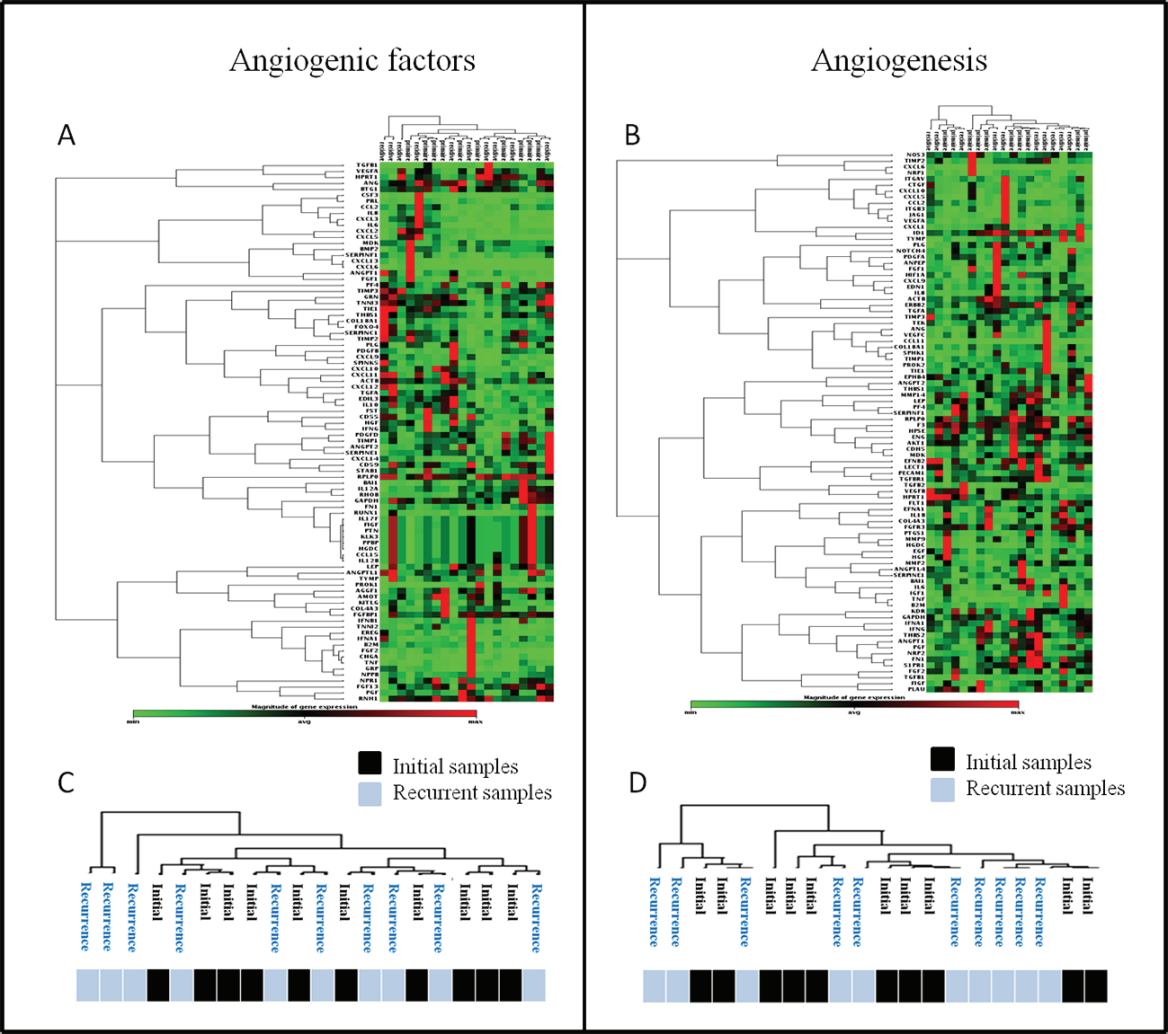
Unsupervised analyses of RT2 profiler PCR arrays **(A)** and **(B)** Unsupervised segregation of samples according to factors expressions for the two types of arrays: angiogenic factors **(A)** and angiogenesis **(B)** arrays. **(C** and **D)** Details of samples clustering after unsupervised segregation of initial and recurrent tumors according to the two types of arrays (C & D): no specific profile of recurrent glioblastoma samples was found.

Screening was performed on the first 10 patients enrolled using the RT^2^ PCR array probe set (Qiagen^®^). Analyses of RT^2^ PCR arrays identified gene expression changes between the samples from initial and recurrent tumors. Among them, *VEGFR2* (*p* = 0.110) and *uPA* (*p* = 0.100) tended to decrease at recurrence, while *CXCL12* (*p* = 0.080) tended to increase. We performed an unsupervised hierarchical clustering analysis (Figure 1A and 1B) which did not identify any specific signature disciminating initial from recurrent angiogenic profiles of paired tumors. Indeed, as showed in Figure 1, no specific clustering of initial versus recurrent samples was observed. (Figure 1C and 1D). Based on these initial results, eight genes were selected for the next step of validation by RT-qPCR: *VEGFA, VEGFR2, VEGFR1, CXCL12, CXCR4, HIF1α, uPA* and *Adrenomedullin (AM)*.

### RNA expression (Figure 2A, Table 2, Supplementary Table 2, Supplementary Figure 1)

**Figure 2:**
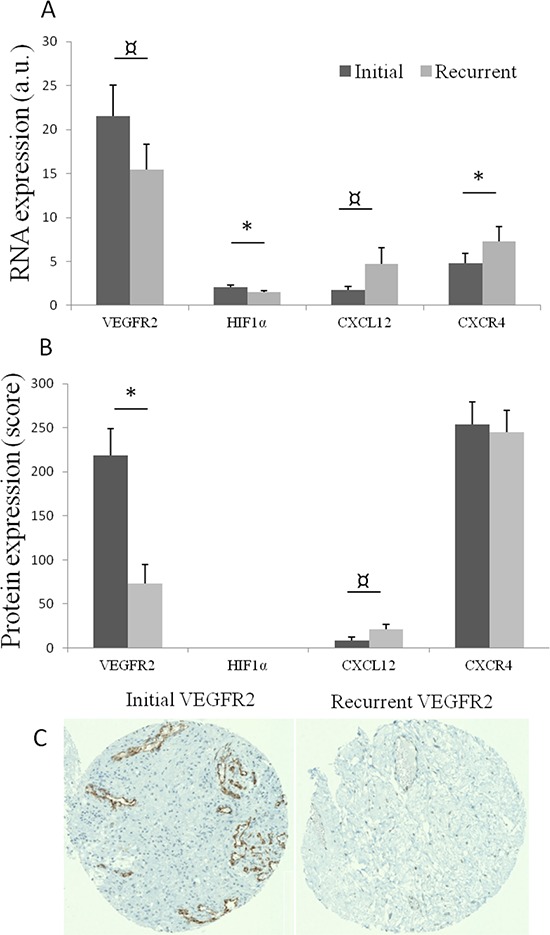
**(A)** Mean RNA expression, with standard error of mean, of VEGFR2, HIF1α, CXCL12 and CXCR4 in initial (dark grey) and recurrent tumors (light grey)a.u.: arbitrary unit: quantitative ratio of tumor expression/control tissue (normal brain) expression. *p < 0.05; *p* < 0.11. **(B)** Mean protein expression (with standard error of mean) of VEGFR2, CXCL12 and CXCR4 in initial and recurrent paired tumors. *p < 0.05; *p* < 0.11. **(C)** An exemple of immunostaining of VEGFR2 in initial and recurrent paired tumors.

To further explore the pathways identified in the previous step, reverse-transcriptase quantitative PCR (RT-qPCR) of the eight selected genes was performed on the entire cohort of 29 patients. RNA expression analyses identified significant changes between initial and recurrent GBM. *CXCR4* expression significantly increased at recurrence (*p* = 0.029), and expression of its ligand *CXCL12* showed a trend toward an increase (*p* = 0.107). In contrast, *HIF1α* expression significantly decreased at recurrence (*p* = 0.009) and there was a trend toward a decrease in *VEGFR2* expression (*p* = 0.081) at recurrence (Figure 2A). Twenty-three patients (80%) presented with both decreases in *VEGFR2-HIF1α* and increases in *CXCL12-CXCR4*, and none presented with neither *VEGFR2- HIF1α* decrease nor *CXCL12-CXCR4* increase. Variations in *CXCL12* expression tended to be correlated to those of *CXCR4* expression (*p* = 0.077) and inversely correlated to variations in *HIF1α* expression (*p* = 0.064).

**Table 2:** Significance of changes in RNA and protein expression between initial diagnosis and recurrence

Markers	qPCR	IHC
VEGFR2	0,081	0,004
HIF1α	0,009	–
CXCL12	0,107	0,096
CXCR4	0,029	0,806
VEGFA	0,534	0,077
VEGFR1	0,683	0,794
AM	0,871	–
uPA	0,387	–

### Immunohistochemistry results (Figure 2B and 2C, Table 2, Supplementary Figure 1)

In order to estimate the protein expression of selected genes, immunohistochemical analyses involving staining for selected proteins on a tissue microarray (TMA) comprising 19 available paired GBM samples were performed. A significant decrease in VEGFR2 expression was observed at recurrence (*p* = 0.004) Figure 2B and 2C, while CXCL12 expression tended to increase at recurrence (*p* = 0.096). No change in CXCR4 immunostaining was observed because CXCR4 was highly expressed in both initial and recurrent tumors.

### Explant culture (Figure 3)

**Figure 3:**
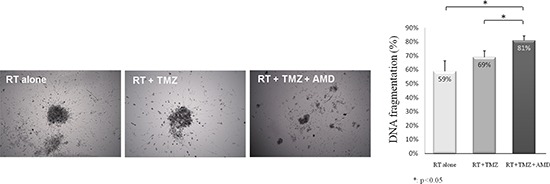
Addition of anti-CXCR4 to radiotherapy and temozolomide increased cell death in GBM explants Representative pictures of explants after 72 h of treatment by radiotherapy (RT) alone, radiotherapy and temozolomide (RT + TMZ), and radiotherapy, temozolomide and 2 μg/mL of anti-CXCR4 AMD3100 (RT + TMZ + AMD). Apoptosis was determined by FACS analysis of DNA fragmentation of propidium iodide-stained nuclei.

The potential anti-tumoral effect of the addition of anti-CXCR4 to the standard of care treatment (radiotherapy and temozolomide) was tested in three distinct explant cultures of GBMs. The addition of anti-CXCR4 was significantly associated with high anti-tumoral effects (Figure 3), decreased explant volume and decreased cell viability. DNA fragmentation increased from 64% to 78% after the addition of anti-CXCR4 to radiotherapy plus temozolomide (*p* = 0.018, Figure 3).

### Clinical impact of RNA expression (Table 3, Figure 4A, 4B, 4C and 4D, Supplementary Table 3)

**Figure 4:**
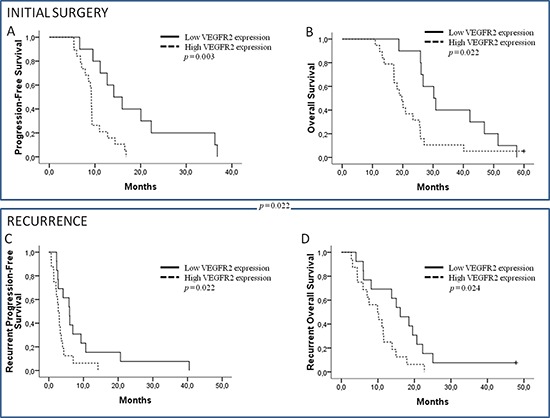
Initial progression-free survival (PFS) and initial overall survival (OS) **(A, B)** According to *VEGFR2* expression on initial diagnosis. Recurrent PFS and OS. **(C, D)** According to *VEGFR2* expression on recurrence.

Median PFS from the initial diagnosis (iniPFS) and OS from the initial diagnosis (iniOS) were 9.4 months (95% CI: 8.9–9.8) and 25.5 months (95% CI: 17.0–34.0), respectively. From recurrence, median PFS (recPFS) and OS (recOS) were 3.3 months (95% CI: 2.3–4.3) and 11.5 months (95% CI: 9.0–13.9), respectively. By multivariate analysis (adjusted by age, recursive partitioning analysis [RPA] classification for PFS, and Karnofsky Performance Status for OS), high *VEGFR2* (Figure 4A, 4B, 4C and 4D) and low *HIF1α* expressions at initial diagnosis were correlated to poor outcomes for iniPFS and iniOS, while low *CXCR4* appeared to only be correlated to worse iniPFS (HR = 0.303). At recurrence, by multivariate analysis, high *VEGFR2* expression was also significantly correlated to both recPFS and recOS, but *HIF1α* and *CXCR4* expressions did not impact outcome at recurrence. Other significant factors were initial *uPA* expression and recurrent *VEGFA* and *VEGFR1* expressions. In this selected population, by univariate analyses, only RPA classification impacted iniPFS (*p* = 0.039) while the effect of other classical prognostic factors was not significant.

**Table 3:** Prognostic value of initial and recurrent factors for progresson-free survival (PFS) and overall survival (OS) at initial diagnosis and recurrence

Factors at initial diagnosis	*p* value	Multivariate (*p*; HR)	*p* value	Multivariate (*p*; HR)
	Initial PFS		Initial OS	
VEGFR2	0,003	0,009* HR = 4,119 (1,432–11,846)	0,02*	0,019* HR = 3,650 (1,233–10,801)
HIF1α	0,009	0,005* HR = 0,275 (0,111–0,679)	0,011	0,012* HR = 0,300 (0,117–0,767)
CXCL12	0,122		0,262	
CXCR4	0,016	0,012* HR = 0,303 (0,119–0,770)	0,290	0,180*
VEGFA	0,190		0,957	
VEGFR1	0,337		0,809	
AM	0,413		0,416	
uPA	0,026	0,032* HR = 0.349 (0,133–0,914)	0,530	
**Factors at recurrence**		**Recurrent PFS**		**Recurrent OS**
VEGFR2	0,022	0,020^¤^ HR = 2,758 (1,177–6,460)	0,024	0,024^¤^ HR = 2,536 (1,133–5,674)
HIF1α	0,391		0,762	
CXCL12	0,589		0,639	
CXCR4	0,762		0,920	
VEGFA	0,032	0,026^¤^ HR = 0,400 (0,178–0,898)	0,226	
VEGFR1	0,050	0,045^¤^ HR = 2,338 (1,019–5,366)	0,052	0,051^¤^
AM	0,080		0,119	
Upa	0,686		0,730	

* Adjusted by Recursive Partitioning Analysis (RPA).

¤ Ajdusted by age and Karnofsky Performans Status (KPS).

### Impact of bevacizumab administration (Supplementary Table 4, Figure 5)

**Figure 5:**
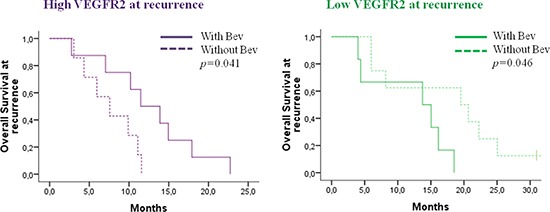
Impact of bevacizumab administration on overall survival for patients with recurrent high or low *VEGFR2* expression, respectively.

In order to explore the potential predictive value of RNA expression for bevacizumab activity, the impact of *VEGFA* and *VEGFR2* expression on patient outcome according to bevacizumab use at recurrence was investigated. No correlations between initial expressions of *VEGFA* and *VEGFR2*, survival and bevacizumab use were observed. At recurrence, *VEGFR2* expression was significantly correlated to the impact of bevacizumab. Patients with high *VEGFR2* expression at recurrence had significant longer OS when using bevacizumab versus other chemotherapy (*p* = 0.041) (Figure 5). In contrast, *VEGFA* expression at recurrence was not correlated to bevacizumab activity.

## DISCUSSION

GBMs are heterogeneous tumors that exhibit temporal and spatial variability. Molecular evolution of these tumors between the initial diagnosis and the inevitable recurrence is poorly documented. Among putative GBM targets, angiogenesis is one of the most attractive pathways [3]; however, to date, no clear data are available on the expression of these factors after radiation therapy, which is one of the oldest anti-angiogenic approaches. In our study – we explored the putative variations in angiogenic expression profiles between the initial and paired recurrent GBM after first-line treatment with radio-chemotherapy. Although we did not identify a molecular angiogenic signature of recurrence, we observed a switch from *VEGFR2*-*HIF1α* to *CXCL12-CXCR4* expression at recurrence, and our results were largely consistent between RNA and protein expression. In addition, we showed that targeting the *CXCL12-CXCR4* pathway in explants of GBM isolated from patients was associated with a pronounced anti-tumor effect.

The angiogenic signature (“angiome”) remains a poorly documented concept [14, 15]. The small number of reported angiogenic profiles and the multiplicity of factors involved in this process may explain the difficulty in identifying a distinct signature between initial and recurrent GBM in our study. Indeed, by unsupervised hierarchical regression analysis we did not find a specific angiogenic profile of recurrent GBM. However, more importantly, we observed a modification of angiogenic pathways at recurrence, with decreasing expression of *VEGFR2* (and *HIF1α* to a lesser extent) in favor of the *CXCL12-CXCR4* pathway in analyses of both RNA and protein. Therefore, the pivotal roles of VEGFR2 and HIF1α in tumor angiogenesis, as widely documented in the literature [16] and observed in our study, appear to be more pronounced at the time of initial diagnosis compared with at recurrence.

The shift that we observed at recurrence in favor of the *CXCL12-CXCR4* pathway could suggest a switch in the tumor vascularization model from angiogenesis to vasculogenesis. In contrast to classical angiogenesis, vasculogenesis corresponds to vessel formation by recruitment of circulating bone marrow-derived cells (BMDC), including myeloid precursor cells, and involves different factors such as hedgehog family factors, angiopoietin-2 or CXCL12 [16]. In pre-clinical cancer models, tumor neo-vascularization was suggested to be preferentially led by vasculogenesis after irradiation, which blocks local angiogenesis [17]. This observation has been reported in various tumor models including breast, lung and brain tumors [17–20]. In these pre-clinical observations based on cultured cells, BMDC appeared to be recruited directly through the CXCL12 expression. Moreover, this process was reported to be, in part, HIF1α induction-independent [21]. In our study, we found similar changes in patients, with a decreased implication of *VEGFR2* at recurrence in favor of the *CXCL12-CXCR4* pathway, for both RNA and protein expressions.

The role of CXCR4 is further supported in our study by *in vitro* data. We evaluated the adjunction of a CXCR4 antagonist on GBM explants isolated from three patients and demonstrated a greater anti-tumor effect with the combination of AMD3100 and radiotherapy plus temozolomide versus radiotherapy and temozolomide alone. The use of GBM explants isolated from patients presented an original advantage to concomitantly analyze tumor cells and their micro-environment, and avoids the bias of cultured cells that lack the typical micro-environment and nude murine models that lack immune systems. A synergic effect of AMD3100 and temozolomide was reported *in vitro* [22]. Moreover, in mouse models of GBM involving the implantation of cultured cells [20, 23] blocking the *CXCL12-CXCR4* pathway in association with radiation therapy appeared to result in prolonged survival [20, 24]. Ideally, the effect of AMD3100 should be tested in explants from both newly diagnosed and recurrent GBM to reinforce our findings.

Finally, comparative analysis of angiogenic markers identified different sub-populations with distinct prognostic and therapeutic responses. First, although *VEGFR2* is known to be over-expressed in GBM, a higher *VEGFR2* expression was consistently correlated to a poor outcome at the initial diagnosis and at recurrence in our study. The prognostic impact of *VEGFR2* expression is currently unclear [25–27]; however, the similar prognostic impact of *VEGFR2* expression for both initial and recurrent survival indicates that it may be substantial. Second, *VEGFR2* expression appears to be correlated to bevacizumab activity when assessed during bevacizumab treatment. *VEGFR2* expression at recurrence, but not at initial diagnosis, was correlated to bevacizumab activity when it was administered at recurrence in our study. Biomarkers of bevacizumab activity are still under investigation, and no clear candidates have emerged to date [28]. VEGFR2 expression, as analyzed by immunohistochemistry, has been reported to be potentially correlated to bevacizumab response, although, in those studies, the analysis of VEGFR2 was generally not performed at the same time as bevacizumab administration [29–31]. When analyzed in plasma, VEGFA and VEGFR2 levels were not predictive of bevacizumab activity, while other biomarkers, such as proteases, may have a role in predicting the benefit of bevacizumab treatment [32]. Because we have shown that the role of the VEGFR2 pathway is more pronounced at the time of initial diagnosis, it would be of interest to explore the correlation of *VEGFR2* RNA expression with benefits conferred by bevacizumab benefit in the upfront setting.

The present study is the first to examine the variation in the molecular profile of angiogenic factors among GBM patients between initial diagnosis and recurrence after SOC (including radiotherapy). We chose to focus on putative angiogenesis targets and their variations over time. Indeed, therapeutic development of anti-angiogenic drugs for GBM patients remains a challenge, and an understanding of the evolution of angiogenic factors in GBM may be helpful in the development of new therapies. However, it should be noticed that the present study was not designed to explore changes after anti-angiogenic exposure; thus, the mechanism of improvement after bevacizumab treatment were beyond the scope of this study. Several limitations to our study should be noted. It is based on a highly selected population of GBM patients who underwent a minimum of two surgeries, which may not fully represent the heterogeneity of GBM biology. However, the OS of our patients was close to those reported for similar patients amenable to a second surgery at recurrence [33, 34]. Our population was monocentric and included a limited number of patients (*n* = 29), although it was one of the largest and homogenous series [10–13], due to the difficulty of obtaining frozen samples of tumors obtained during both initial and recurrent surgeries. Additional studies are required to confirm our study.

## PATIENTS AND METHODS

### Tumor samples

Patients who underwent a minimum of two surgical resections (at initial diagnosis and first recurrence) were retrospectively identified from the authors' tumor bank (Assistance Publique–Hôpitaux de Marseille [APHM], Timone, Marseille, France). GBMs were diagnosed according to the 2007 World Health Organization classifications [35]. All frozen samples were stored in the APHM Tumor Bank (authorization number 2013–1786). Histological review of the frozen samples (DFB) confirmed the neoplastic nature of the tissue and demonstrated lack of normal residual tissue in samples used for RT-qPCR techniques. Genomic DNA was systematically extracted; methylation of the MGMT promoter and *IDH1/2* mutation were evaluated as previously described [12, 36]. Tumor specimens were obtained after written consent and according to a protocol approved by the local institutional review board and ethics committee. The present study was conducted in accordance with the declaration of Helsinki.

### Treatment and clinical follow-up

First-line treatment consisted of radiotherapy (60 Gy in 30 fractions) with concomitant temozolomide (75 mg/m^2^ daily) followed by six cycles of adjuvant temozolomide (150 mg/m^2^ up to 200 mg/m^2^ for five days every 28 days) for 27 of 29 patients. Two patients received BCNU (150 mg/m^2^ on day 1 every 6 weeks) instead of temozolomide. At first recurrence, all patients underwent a second surgery. Patients with early progression within three months after radio-chemotherapy were excluded in order to avoid potential pseudo-progression. Clinical follow-up was performed every four weeks and magnetic resonance imaging every eight weeks by a senior physician. Disease evaluation was performed according to either the Macdonald and RANO [37] criteria, according to the date of evaluation. All patients experienced progression and only one patient was still alive at the last contact, with a follow-up of 60 months.

### RNA extraction

Total RNA was extracted using TriPrep NucleoSpin^®^ (Macherey–Nagel, Germany), according to the manufacturer's instructions. RNA was analyzed on the Nanodrop spectrophotometer and Agilent 2100 bioanalyzer (Agilent Technologies, Massy, France) for quantitative and qualitative controls. Only samples with no evidence of ribosomal peak degradation and RIN values ranging between 6.0 and 10.0 were considered to be high-quality intact RNA [38].

### Reverse transcription

Total DNA-free RNA (1 μg) was reverse-transcribed into complementary DNA using 1 μg of random hexamers (Roche^®^) and Moloney murine leukemia virus reverse transcriptase (MMLV-RT), Invitrogen^®^) as recommended by the manufacturer.

### RT^2^ PCR array

First, screening was processed on ten patients with the two types of probe sets RT^2^ profiler PCR Arrays (Qiagen^®^): angiogenesis (PAHS-024Z) and angiogenic growth factors (PAHS-072Z) arrays. Each arrays allow the expression analysis of 84 genes, including 20 genes common, to both arrays, leading to the totally evaluation of 148 distinct genes. RNAs were first reversed transcribed into cDNA and then were processed using the LightCycler 480 (Roche Applied Science) as recommended by the manufacturer. Each array was composed by 84 different primers related to angiogenesis and angiogenic growth factor and five keeping house genes. Comparative expressions were computed using the specific Qiagen^®^ software. Gene expression changes with *p* values < 0.15 were selected for the next step.

### Real-time quantitative PCR (RT-qPCR)

All 29 patients (= 58 samples) were processed for the RT-qPCR experiment using a LightCycler 480 (Roche Applied Science) and the LightCycler 480 SYBR Green I Master Mix (Roche Applied Science). All experiences were performed in triplicate. The relative expression ratio of the target messenger RNA and reference RNA (18S, glyceraldehyde-3-phosphate dehydrogenase, β-actin) was calculated using qPCR efficiencies and the crossing point (Cp) deviation of a tumor sample versus normal adult human brain (Agilent Technologies) used as a control tissue [39, 40]. Forward and reverse primers for each gene are listed in Supplementary Table 1.

### Immunohistochemistry

Immunohistochemical analysis was performed on tissue microarrays (TMA) that were constructed from routinely processed formalin-fixed paraffin-embedded tumor material. Areas of viable and representative tumor following review of all blocks were marked by a pathologist (DFB) before inclusion in the TMA (3 × 0.6 mm cores for each tumor). A Benchmark Ventana autostainer (Ventana Medical Systems SA, Illkirch, France) was used for detection and TMA slides were simultaneously immunostained to avoid inter-manipulation variability. Slides immunostained for VEGFA (Goat IgG, R&D Systems, Lille, France, Europe), VEGFR1 (Goat IgG, R&D Systems, Europe), VEGFR2 (clone SSB11, Rabbit IgG, Cell Signalling), CXCL12 (clone C-19, Goat IgG, Santa Cruz, Biotechnology Inc, Heidelberg, Germany) and CXCR4 (clone 44716, Mouse IgG2B, R&D Systems, Europe) were scored by a pathologist (DFB).

### Explant culture of GBM samples isolated from patients

Three GBM tissue samples were collected after surgery and placed in Dulbecco's modified Eagle's medium (DMEM) supplemented with 0.5% fetal calf serum (FCS), 1% penicillin-streptomycin and 1% sodium pyruvate (Gibco-Invitrogen, Cergy Pontoise). Tissues were cut into 500 μm pieces in DMEM + 10% FCS, and plated on 12-well plates precoated with poly-(L)-lysine (10 μg/mL; Sigma) for cell death analysis. Medium was supplemented with 0.4% methycellulose (Sigma). Explant cultures were then incubated at 37°C in a 5% CO_2_ and 95% air atmosphere. After 72 h of culture, explants were treated with 100 μM temozolomide (Sigma) and/or 2 μg/mL AMD3100 (Sigma) followed by 6 Gy of irradiation. After 72 h of treatment, explants were dissociated and fluorescence-activated cell-sorting (FACSCalibur, Becton Dickinson, Le Pont-De-Claix, France) analysis of DNA fragmentation of propidium iodide-stained nuclei was performed, as previously described [41].

### Statistical analyses

Data are expressed as mean ± standard error. Statistical analysis was performed using Student's *t-*test and the Wilcoxon test. The Mann–Whitney *U*-test was used to compare quantitative variables. Correlations were analyzed using the Spearman correlation. OS from the initial diagnosis (iniOS) was defined to be the time from initial diagnosis to death from any cause, censored at the date of last contact. OS from the first recurrence (recOS) was defined to be the time from second surgery to death from any cause, censored at the date of last contact. Initial PFS (iniPFS) was the time from initial diagnosis to documented progression or death, censored at the date of the last documented disease evaluation. Recurrent PFS (recPFS) was the time from second surgery to progression or death. The Kaplan–Meier method was used to estimate survival distribution. Log-rank tests were used for univariate comparisons and Cox proportional hazard regression models were used to estimate the hazard ratio (HR) in multivariate analyses. ROC analyses were performed determine the optimal cut-off for low and high RNA expression, considering the OS as a categorical variable with the median OS as a cut-off. In cases of nonsignificant correlation between RNA expression and survival in continuous variables, the median expression was arbitrarily chosen as the cut-off to define low and high RNA expression. For the analysis of the explants obtained from GBM samples from patients, the Wilcoxon test was used. All reported *p* values are two-sided, and *p* < 0.05 was again considered to be statistically significant. Statistical analyses were performed using SPSS PASW statistics 22.0.

## CONCLUSION

Angiogenesis remains a complex feature of GBM that changes over time, even without exposure to antiangiogenic treatment. Recurrence of GBM after chemo-radiation could be associated with a switch of angiogenic pattern from *VEGFR2-HIF1α* to *CXCL12-CXCR4* pathway, leading to new perspectives in angiogenic treatment.

## SUPPLEMENTAL FIGURE AND TABLES


