# Supplementary material for: Recurrence of glioblastoma after radio-chemotherapy is associated with an angiogenic switch to the CXCL12-CXCR4 pathway
Source: Oncotarget. 2015 Mar 25;6(13):11664–75. doi: 10.18632/oncotarget.3256 (PMC4484484; doi:10.18632/oncotarget.3256)
Supplement: Supplementary file 1 [file oncotarget-06-11664-s001.pdf]

SUPPLEMENTARY FIGURE AND TABLES

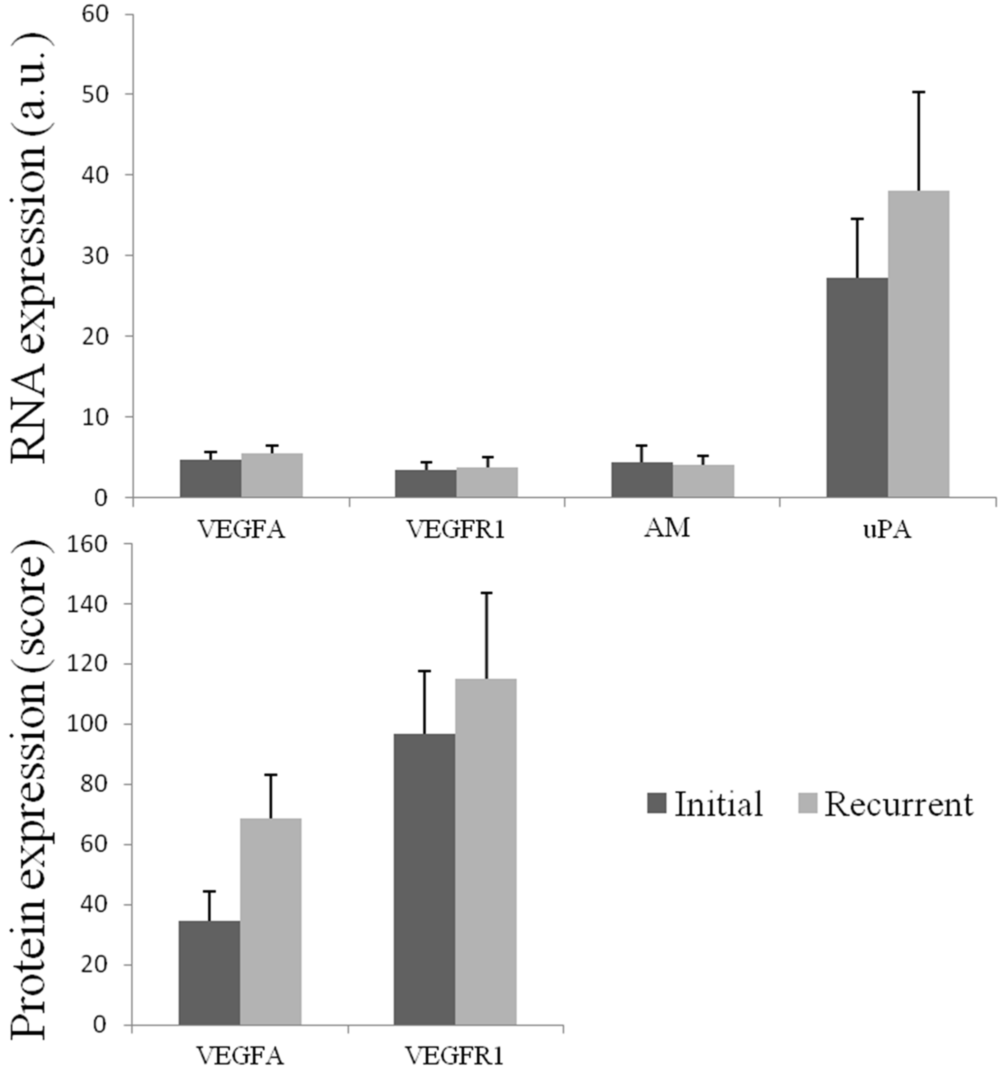

Supplementary Figure 1: Mean RNA expression, with standard error of mean, of *VEGFA*, *VEGFR1*, *AM* and *uPA* in initial (dark grey) and recurrent tumors (light grey). a.u.: arbitrary unit: quantitative ratio of tumor expression/control tissue (normal brain) expression. Protein expression of VEGFA and VEGFR1 in initial and recurrent paired tumors.

Supplementary Table 1: Primer sequences and conditions

Gene Name	Sequences	Conditions
VEGFA	S : 5′-AGGAGGAGGGCAGAATCATCA-3′<br>AS : 5′-AGGGTCTCGATTGGATGGC-3′	95°C 15s ; 66°C 30s
VEGFR1	S : 5′-GAACCCCGATTATGTGAGAAA-3′<br>AS : 5′-GATAGATTCGGGAGCCATCC-3′	95°C 15s ; 65°C 30s
VEGFR2	S : 5′-AACATTTGGGAAATCTCTTGC-3′<br>AS : 5′-GGAAGAACAATGTAGTCTTT-3′	95°C 15s ; 65°C 30s
AM	S : 5′-TGCCCAGACCCTTATTCGG-3′<br>AS : 5′-AGTTGTTCATGCTCTGGCGG-3′	95°C 15s ; 67°C 30s
CXCL12	S : 5′-CGATTCTTCGAAAGCCATGT-3′<br>AS : 5′-TTGTCTGTTGTTGTTCTTCAGC-3′	95°C 15s ; 65°C 30s
CXCR4	S : 5′-AGGATATAATGAAGTCACTATGGGAAA-3′<br>AS : 5′-AAGGGCACAAGAGAATTAATGTAGA-3′	95°C 15s ; 62°C 30s
HIF1 α	S : 5′-AAGGAACCTGATGCTTTAACTTTG-3′<br>AS : 5′-TGGTCATCAGTTTCTGTGTCG-3′	95°C 15s ; 65°C 30s
UPA	S : 5′-CTTAACTCCAACACGCAAGGGG-3′<br>AS : 5′-AGCTTGTGCCAAACTGGGGATC-3′	95°C 15s ; 65°C 30s
GAPDH	S : 5′-CAAATTCCATGGCACCGTC-3′<br>AS : 5′-CCCACTTGATTTTGGAGGGA-3′	95°C 15s ; 65°C 30s
β-ACTINE	S : 5′-CCACACTGTGCCCATCTACG-3′<br>AS :5′-AGGATCTTCATGAGGTAGTCAGTCAG-3′	95°C 15s ; 65°C 30s
18S	S : 5′-CTACCACATCCAAGGAAGGCA-3′<br>AS : 5′-TTTTTCGTCACTACCTCCCCG-3′	95°C 15s ; 67°C 30s

Supplementary Table 2: Mean RNA expressions and standard error of the mean at initial diagnosis and recurrence

	Initial diagnosis		Recidive	
	Mean	*Std Error*	Mean	*Std Error*
VEGFR2	2154	*354*	1547	*288*
HIF1α	208	*25*	154	*17*
CXCL12	179	*33*	469	*185*
CXCR4	480	*112*	726	*168*
VEGFA	467	*103*	557	*93*
VEGFR1	348	*91*	378	*125*
ADM	449	*195*	414	*109*
uPA	2735	*724*	3803	*1235*

Supplementary Table 3: Continuous correlation between RNA expression and median iniOS. Cut-off determined by receiver operating characteristic analysis

Factor	Initial Median OS (*p value*)	Cut-off
CXCR4	0,130	
CXCL12	0,098	70,6
uPA	0,058	250,7
HIF1α	0,156	
VEGFA	0,354	
VEGFR2	0,009	792,6
VEGFR1	0,093	169,2
AM	0,252	

Supplementary Table 4: Potential predictive value of RNA expression levels for bevacizumab activity at recurrence

Markers for bevacizumab activity prediction	Recurrent PFS	Recurrent OS
**Initial VEGFA**		
High	0,783	0,413
Low	0,674	0,251
**Recurrent VEGFA**		
High	0,234	0,215
Low	0,442	0,442
**Initial VEGFR2**		
High	0,934	0,941
Low	0,382	0,240
**Recurrent VEGFR2**		
High	0,358	0,041
Low	0,150	0,046
